# Tranexamic Acid Reduces Transfusion Rates After Modular Hemiarthroplasty for Pathological Femoral Fractures: A Retrospective Study

**DOI:** 10.1155/aort/3173784

**Published:** 2025-10-30

**Authors:** Piotr Biega, Grzegorz Guzik

**Affiliations:** ^1^ Department of Physics and Medical Engineering, Rzeszów University of Technology, Rzeszów, Poland, prz.edu.pl; ^2^ Orthopaedic Oncology Department, Subcarpathian Oncology Centre, Brzozów, Poland

## Abstract

**Background:**

Fractures of the femur are one of the major complications of solid tumor metastasis to bone. Tumor resection with reconstruction using modular prostheses achieves optimal local tumor control, pain reduction, and improved patient function and quality of life. Unfortunately, the surgical treatment of femoral fractures with prostheses is associated with high blood loss, requiring the transfusion of blood products. Blood transfusion carries several risks, including immune suppression and a higher risk of postoperative infection, deterioration of functional outcomes, and prolonged hospital stays. An inexpensive and effective way to reduce blood loss and the number of transfusions is tranexamic acid (TXA).

**Methodology:**

In our study, we analyzed 82 cases of patients treated with resectional prostheses for pathological fractures between 2017 and 2021. The operations of 42 patients were performed between 2017 and 2019 without the use of TXA. Another 38 patients were operated on in 2020‐2021 with the use of TXA prophylaxis. Total blood loss (TBL), hemoglobin drop, number of transfusions, and adverse thromboembolic events were evaluated.

**Results:**

After surgery, patients who received TXA had higher hemoglobin levels on the first day than those in the control group (0.99 g/dL *p* = 0.03), and the blood loss was reduced by 217 mL in the TXA group (*p* = 0.09). Transfusion rate was reduced from 43% to 17% (*p* = 0.04). No differences were seen in the number of complications.

**Conclusion:**

TXA significantly reduced transfusion rates and hemoglobin drop, without increasing complications.

**Trial Registration:**

ClinicalTrials.gov identifier: NCT06244498

## 1. Background

Cancer is one of the greatest challenges facing the healthcare system today. The skeletal system is the third most common site of metastasis for solid tumors, after the lungs and liver. Metastases occur most commonly in the spine, with the proximal femur being affected three times less frequently. In the femur, metastatic lesions are found in the neck (50%), the intertrochanteric region (20%), and the subtrochanteric region (30%). Only 10% of patients with disseminated cancer experience pathological fractures [1, 2].

Femoral fractures are associated with substantial blood loss, ranging from 500 to 1500 mL. In 39%–69% of cases, patients develop anemia, which may require blood transfusion [3]. Blood transfusions may lead to immune system dysfunction and posttransfusion reactions, increasing the risk of infections, prolonged hospital stays, and decreased quality of life [4]. Several blood preservation techniques have been developed, including hypotensive anesthesia, regional anesthesia, autotransfusion kits, and drugs that inhibit fibrinolysis [5].

Tranexamic acid (TXA) reversibly and effectively inhibits plasminogen activation [6]. Evidence suggests that TXA reduces blood loss, increases hemoglobin levels (HGLs), and decreases the need for blood transfusions in patients undergoing both elective and traumatic orthopedic procedures [7, 8]. However, the incidence of perioperative venous thromboembolic events (VTEs) following TXA administration remains uncertain [9]. Moreover, cancer cells secrete procoagulant factors, increasing the risk of VTE by approximately sixfold compared to the general population [10]. Consequently, patients with pathological fractures are typically excluded from clinical studies due to their elevated risk of VTE [10–16]. To date, only two studies have investigated the use of TXA in this high‐risk patient population [17, 18]. This study assesses the safety and efficacy of TXA in patients treated for pathological femoral fractures using modular hemiarthroplasty.

## 2. Methodology

The medical records of patients who underwent resection modular hemiarthroplasty for metastatic tumors of the proximal femur between 2017 and 2021 were analyzed in this study. All patients received a modular prosthesis and were administered thromboprophylaxis with enoxaparin (40 mg once daily) throughout their hospital stay. The same experienced staff performed the operations for all cases.

Inclusion criteria consisted of proximal femur fractures in the cervicocephalic and trochanteric segments (31A, B, C) or subtrochanteric segments (32A1.1/32A2.1/32A3.1 and 32B1.1/32B2.1/32B3.1), according to the AO classification, resulting from metastatic lesions, with an expected survival of more than three months. Life expectancy was estimated by a multidisciplinary team comprising medical oncologists, radiologists, radiotherapists, and orthopedic surgeons.

Patients with primary neoplasms of the proximal femur or metastatic lesions treated using a conventional stem, long‐stem arthroplasty, intramedullary nailing, or other osteosynthesis methods were excluded from the study.

To evaluate the effectiveness of the intervention, we reviewed the records of patients who underwent surgery between 2017 and 2019, when TXA was not administered (control group). Since 2000, all patients have received TXA at a dose of 1.0 g during induction of anesthesia and again immediately after surgery (study group). As no standardized regimen has been established, the dosing schemes were selected based on the preferences of the surgical team [7].

On the first postoperative day, a complete blood cell count (CBCC) was performed. For patients with HGL below 9 g/dL, an additional CBCC was conducted. Red blood cell transfusions were administered to patients whose HGL dropped below 8 g/dL. The decision to transfuse was made by the attending physician, based on the patient’s clinical condition and CBCC results.

Parameters including age, sex, operative duration, resection size, pre‐ and postoperative HGL, total blood loss (TBL), and adverse events were evaluated. Adverse events were defined as cardiovascular events—such as stroke, myocardial infarction, arrhythmias, deep vein thrombosis, and pulmonary embolism—as well as other complications, including acute renal insufficiency, wound infection, and persistent bleeding.

Gross TBL was estimated using the formula:

(1) Total blood loss=blood volumeBV×Hct_pre−Hct_postHct_avg.

Total circulating blood volume was calculated using the Nadler formula:

(2) BVmL=k1×heightm+k2×weightkg+k3.

• For males: *k*_1_ = 0.3669, *k*_2_ = 0.03219, *k*_3_ = 0.6041.
• For females: *k*_1_ = 0.3561, *k*_2_ = 0.03308, *k*_3_ = 0.1833.

Technical abbreviations were defined upon first use. A nonblinded assessment of outcomes was performed. Listwise deletion technique was selected for managing missing data (8 records). All variables within the groups were assessed for distribution. For normally distributed variables, means and standard deviations were reported; for nonnormally distributed variables, medians along with upper and lower quartiles were provided.

Independent variables were analyzed using Student′s *t*‐test for continuous data and the chi‐squared test for categorical data. Logistic regression models were used to evaluate the effectiveness of TXA, adjusting for variables that influenced hemoglobin, hematocrit, and blood loss in univariate analyses.

All statistical analyses were two‐tailed, and *p* values of < 0.05 were considered statistically significant. All statistical analyses were performed using *Statistica* Version 13 (TIBCO Software Inc.).

## 3. Result

**Figure 1:**
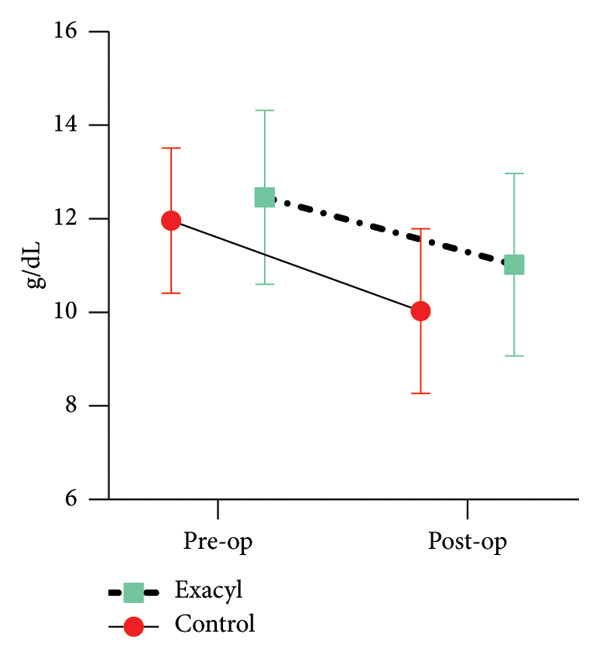
Hemoglobin level.

The study included 110 cases treated between 2017 and 2021; however, 22 patients were excluded from subsequent analysis due to threatened fractures, intraoperative blood transfusions, or fracture fixation methods that precluded the use of resectable prostheses. The final analysis included a series of 82 cases. Of these patients, 38 received TXA, while the control group comprised 42 patients (Table 1). The mean age of the participants was 70.5 years (±10.7), with females accounting for 54% of the sample. Hypervascular tumors were observed in 22% of patients (18% in the control group vs. 24% in the TXA group). The mean duration of the surgical procedure was 90 min (±30), and the mean resection length was 13 cm (range, 10–14 cm). Preoperative HGL levels had a mean value of 11.7 g/dL (±1.79) and decreased to 10.5 g/dL (±1.86) postoperatively. Baseline hemoglobin values did not differ significantly between groups. However, on the first postoperative day, HGL tended to be higher in the TXA group by 0.99 g/dL compared to the control group (*p* = 0.03; 95% CI = 0.84–1.15) (Figure 1).

**Table 1:** Study group demographics and hematological status.

Group with number	TXA *N* = 38	Control *N* = 42
Mean age (years ± SD)	70 (7)	71 (10)
Women (%)	54	46
Mean operation time (min ± SD)	89 (38)	92 (29)
The median resection length (cm lower‐upper quartiles)	14.8 (11.5–20)	12.4 (10–14)
Hypervascular tumors (%)	24	18
Mean HGL presurgery (g/dL ± SD)	12.5 (1.85)	11.4 (1.54)
Mean HGL postsurgery (g/dL ± SD)^∗^	11.03 (1.94)	10.02 (1.75)
Mean HT presurgery (% ± SD)	37 (4.3)	36 (4.8)
Mean HT postsurgery (% ± SD)	32 (4.2)	30 (6.2)
Mean TBL (mL ± SD)	885 (454)	1102 (627)
Blood transfusion (yes)^∗^	17	43

*Note:* Data are presented as mean ± standard deviation of the mean, median (with lower and upper quartiles), or number (percentage), where appropriate.

Abbreviations: HGL = hemoglobin level, HT = hematocrit, RR = relative risk, TBL = total blood lost, and TXA = tranexamic acid.

^∗^ *p* < 0.05.

The estimated TBL was 1102 mL in the control group and 885 mL in the TXA group. However, this difference did not reach statistical significance (*p* = 0.09; 95% CI = 747–1022). The incidence of postoperative blood transfusion was significantly lower in the TXA group (17%) compared to the control group (43%; *p* = 0.04). Patients requiring transfusion exhibited lower baseline HGL than those who did not (11.4 vs. 12.5 g/dL; *p* = 0.14; 95% CI = 0.22–0.97).

**Figure 2:**
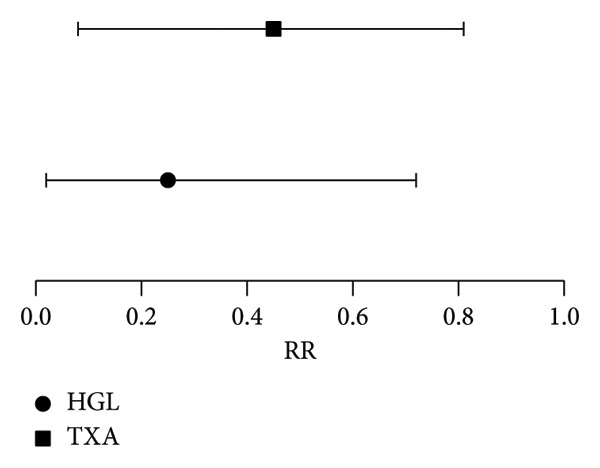
Relative risk for transfusion.

Multivariate logistic regression analysis identified two independent predictors for a reduced risk of transfusion: higher baseline HGL (RR = 0.25; 95% CI = 0.03–0.72; *p* = 0.02) and administration of TXA (RR = 0.45; 95% CI = 0.08–0.81; *p* = 0.01) (Figure 2). No significant correlations were observed between resection length and hemoglobin drop or transfusion requirements.

No significant differences in postoperative complications were observed between the two groups. No postoperative mortalities were noted within 30 days. Three cases of VTE were reported—one in the TXA group and two in the control group. There were no recorded incidences of arrhythmias, acute renal insufficiency, or wound infections.

## 4. Discussion

TXA was administered at a dose of 1.0 g twice, once after skin incision and again before surgical wound closure. This regimen has been shown to significantly reduce the transfusion rate and HGL drop in patients undergoing resectional prosthetic surgery for metastatic femoral tumors.

The efficacy of TXA in reducing perioperative blood loss and transfusion requirements has been confirmed in numerous studies, including randomized population‐based trials and meta‐analyses [7, 8, 19–21]. In the context of surgical treatment for pertrochanteric fractures, TXA has been shown to reduce TBL by 199–266 mL, HGL drop by approximately 0.46 g/dL, and the need for transfusion by 17%–26%. However, many of these studies included a heterogeneous population of femoral fractures treated with varying methods, including internal fixation and prosthetic implantation. A recent meta‐analysis involving 54,843 patients treated exclusively with femoral prostheses demonstrated more pronounced benefits of TXA. In this subgroup, TXA reduced hemoglobin decline by 0.83 g/dL and decreased transfusion requirements by 52% [22].

Nevertheless, Spitler et al. indicated less favorable outcomes of TXA in patients with pelvic fractures [23]. In addition, other authors have reported no significant reduction in transfusion rates among patients undergoing hemiarthroplasty for metastatic lesions [18]. However, the observed ineffectiveness of TXA in these studies may be attributable to the inclusion of both pathological and impending fractures, potentially diminishing its therapeutic effect. In contrast, the present study included patients only with confirmed pathological fractures. Within this more homogeneous cohort, administration of TXA was associated with a notable reduction in transfusion rates (17% vs. 43% *p* = 0.04). In addition, the observed difference in postoperative HGL drop was found to be 0.99 g/dL higher in the control group (*p* = 0.03). Nevertheless, TBL was observed to be lower in the TXA group; however, this difference did not reach statistical significance (*p* = 0.09; 95% CI, 747–1022). This tendency may result to surgery following pathological fractures, which were associated with significantly reduced preoperative hematocrit levels. Additionally, solid malignancies and severe trauma have been shown to trigger a hyperfibrinolysis, which may contribute to the diminished efficacy of TXA in these patient populations [24, 25]. Moreover, in patients with hypervascular tumors (22% in our study), TXA does not significantly reduce blood loss [26].

The administration of antifibrinolytic agents such as TXA raises significant safety concerns due to a potential increased risk of VTE. A large retrospective study involving over 21,000 trauma patients reported that administration of TXA was associated with at least a threefold increase in the incidence of VTE [27]. A randomized trial by Zufferey et al.​ in patients undergoing surgery for femoral fractures demonstrated a threefold increase in the risk of VTE, including distant events such as myocardial infarction and stroke [28]. Furthermore, Emar et al. reported laboratory evidence indicating an increased thrombotic tendency in patients receiving TXA [29], while Xie et al. described cases of cardiogenic shock attributed to thrombus formation following TXA administration [30]. Nevertheless, most randomized controlled trials (RCTs) and meta‐analyses have not demonstrated an increased risk of thromboembolism associated with the use of TXA [30, 31]. This apparent discrepancy may be explained by the increased activity of the fibrinolytic pathway observed in patients following bone fractures—which can persist for up to 24 h postinjury—and by the localized action of TXA at the site of injury [32]. Moreover, the exclusion of patients with a history of medical conditions, including prior thrombosis, myocardial infarction, stroke, or cancer, is a common practice in RCTs. It is noteworthy that TXA was administered to a mere 3% of patients treated for proximal femoral injuries [21]. In the present study population, no VTEs were observed. Unfortunately, long‐term follow‐up data regarding vascular complications were not available in our study. Moreover, data from a large database containing 4497 records of patients undergoing surgical fixation for neoplastic pathologic fractures of the lower extremities show that TXA reduces the risk of VTE (OR 0.33) without increasing the risk of other complications at 90‐day follow‐up [17]. Nevertheless, it remains essential for future studies to account for the substantial coagulation abnormalities commonly observed in oncology patients [33].

Developing optimal dosing regimens for TXA remains a clinical challenge. Ideally, dosing should be individualized based on the patient’s coagulation profile to minimize the risk of thromboembolic complications while preserving its hemostatic benefits. However, the optimal timing, dosage, frequency, and route of administration are yet to be clearly established.

In vitro studies suggest that a serum concentration of 10 mg/L is sufficient to inhibit fibrinolysis by approximately 80% [34].

In studies evaluating TXA in periarticular fracture management, doses typically ranged from 10–15 mg/kg IV or 2‐3 g IM, administered once or twice. The timing of administration varied, with TXA given either shortly before the surgical procedure, between 10 and 30 min after injury, or up to 12 h postinjury. A second dose was often given immediately postoperatively, 3–8 h after the initial dose, or as a continuous infusion of 1–5 mg/kg/h during surgery or extending up to 24 h [19, 21, 35].

TXA demonstrated efficacy in reducing blood loss across these studies, regardless of dose or administration route. Notably, one of the few trials comparing different dosing strategies found the greatest hemostatic benefit with a low‐dose bolus of 10 mg/kg followed by a continuous infusion of 1 mg/kg/h, compared to single bolus or high‐dose infusions [36].

Trauma patients, unlike those undergoing elective surgery, experience two distinct injury phases: the initial trauma during fracture and a subsequent physiological insult at the time of surgery. Findings from the landmark CRASH‐2 trial underscore the importance of administering TXA as early as possible after trauma, demonstrating the greatest efficacy when given immediately postinjury [28]. However, Jiganti et al. raised concerns regarding the value of additional perioperative dosing in patients who had already received an initial dose following injury [37]. In contrast, Cui et al. reported improved outcomes when administering 1 g of TXA daily until surgery was performed [37], while Kahan et al. found that four‐dose regimens—two administered postinjury and two perioperatively—were more effective than one‐ to three‐dose protocols [16].

In our study, two 1 g doses of TXA were administered—one prior to surgery and one following skin closure. As previously discussed, individualized TXA dosing may optimize clinical outcomes while minimizing thromboembolic risk. Conversely, high‐dose TXA regimens may be appropriate in palliative oncology settings to control bleeding, and emerging evidence also suggests a potential anticancer effect of TXA [38, 39]. Accordingly, future research should prioritize the development of optimal dosing strategies for TXA use in patients with malignancy.

While this study was conducted with care, it has its limitations. Firstly, the sample size is relatively small and the study is single center. A large‐scale study is necessary to assess drug safety unequivocally in this population. Secondly, this study only considered a short follow‐up period and only measured the HGL on the first postoperative day and thromboembolic complications were assessed in short term—hospital discharge. The lack of long‐term follow‐up may limit the ability to fully evaluate the clinical efficacy and safety of the treatment. Finally, as this study only employed one surgical technique and fixed dose protocol, other treatment techniques and dose regimens will require more in‐depth investigation. Lastly, additional factors should be included in future analysis like fibrinogen level and thrombelastography.

## 5. Conclusion

Orthopedic surgeons should consider the utilization of IV TXA in patients treated surgically for neoplastic pathologic fractures of the lower extremity for reducing perioperative transfusion requirements.

**Nomenclature**
BV: Blood volume
CBCC: Complete blood cell count
HGL: Hemoglobin level
RCTs: Randomized controlled trials
TBL: Total blood loss
TXA: Tranexamic acid
VTEs: Venous thromboembolic events

## Ethics Statement

The research has been performed in accordance with the Declaration of Helsinki. As this retrospective analysis consists of anonymized clinical routine data, the Research Ethics Committee deems the application for and issue of ethics approval not necessary. All data were anonymized before the authors started the research (had access to the data). All patients gave written consent to the use of data for research. Medical Ethics Committee of the ORL in Krakow, ul Krupnicza 11a 31‐123 Cracov, Email address: bioetyka@oilkrakow.org.pl Phone: (12) 619 17 12.

## Consent

All patients gave written consent to the use of data for research.

## Disclosure

All authors have read and approved the final manuscript.

## Conflicts of Interest

The authors declare no conflicts of interest.

## Author Contributions

Piotr Biega analyzed and interpreted the patient data, performed statistical analysis, and was a major contributor in writing the manuscript. Grzegorz Guzik supervised the project and revised it.

## Funding

This research received no specific grant from any funding agency in the public, commercial, or not‐for‐profit sectors.

## Data Availability

The datasets used and/or analyzed during the current study are available from the corresponding author on reasonable request.
